# Editorial: Artificial intelligence: A step forward in biomarker discovery and integration towards improved cancer diagnosis and treatment

**DOI:** 10.3389/fonc.2023.1161118

**Published:** 2023-03-31

**Authors:** Mónica Hebe Vazquez-Levin, Jaume Reventos, George Zaki

**Affiliations:** ^1^ Instituto de Biología y Medicina Experimental (IBYME), Consejo Nacional de Investigaciones Científicas y Técnicas de Argentina (CONICET) Fundación IBYME (FIBYME), Buenos Aires, Argentina; ^2^ Institut d’Investigacio Biomedica de Bellvitge (IDIBELL) and Universitat Internacional de Catalunya, Barcelona, Spain; ^3^ Frederick National Laboratory for Cancer Research, National Cancer Institute at Frederick (NIH), Frederick, MD, United States

**Keywords:** cancer, artificial intelligence, machine learning, digital twin, precision medicine

In cancer, a biomarker refers to a substance or process indicative of the presence of cancer in the body. However, the idea of “one-molecule (or process) marker” indicated by its presence, and the existence of an undergoing transforming cancer process is currently a utopia. During the past decade, there has been a fundamental shift in cancer research and clinical decision-making, moving from qualitative data to quantitative digital data. A large wealth of cancer biomarkers and images has come from research laboratories and clinical institutions worldwide. Moreover, the major bulk of information has arisen from genomics, proteomics, metabolomics, and other omics, but also from oncology clinics, imaging, epidemiology and more. Artificial Intelligence (AI) is a unique technology that is able to combine all the above, and particularly suited to establish novel therapies and predictive models of drug response (1, 2). The combination of several biomarkers, by means of Machine Learning (ML) algorithms, would reach unprecedented conclusions in diagnosis, prediction and general decision making of novel anticancer therapies (3–5). In addition, the multimodal temporal data collected from patients with cancers can feed to initialize and track a Digital Twin to experiment with multiple possible treatments *in silico*.

This Research Topic has gathered 10 selected contributions in the area of ML tools, Deep Learning and Cancer Digital Twin technologies in the field of Precision Oncology, and contains one review, one minireview and eight original contributions.

The review paper by Asada et al. emphasizes the relevance of Precision Oncology and the integration of whole genome sequencing analysis, epigenome analyses and the use of ML, and opens a discussion about future perspectives in the field.

Networks of cellular systems and arrays of biological models described by ordinary and partial differential equations were developed in the last decades towards a better understanding of biological systems. Mertins minireview describes the use of ML algorithms to analyze computational dynamic ordinary differential equation models in combination with omics data, towards the discovery of novel biomarkers and novel molecular targets.

Tumor cell heterogeneity has been for many years a distortion factor in the interpretation of cellular and molecular findings in oncology. Tumor degree of purity is playing an important role in optimizing the correlation of the research findings with therapeutic anticancer strategies. By using Random Forest ML, Yang et al. were able to assess tumor purity in children CNS tumors, which will imply genomic, biological and clinical implications.

More than 60% of cervical cancers are caused by Human Papilloma Virus (HPV) 16 genotype, classified into lineages A, B, C, and D. In their contribution, Asensio-Puig et al. report the development of a Random Forest-based new model to assess HPV16 lineage. Authors highlight that their model is 40 times faster than current assessment done with Maximum Likelihood Tree, which requires a manual annotation and cannot assess poorly sequenced samples.

The work by Chang et al. proposes a novel ML predictive model utilizing a three-Dimensional Convolutional Neural Network (3D-CNN) to predict the presence of lymph node metastasis and the postoperative positive margin status based on preoperative CT scans. Their report provides a proof of concept for the preoperatively use of radiomics and 3D-CNN deep learning framework to improve the prediction of positive resection margins as well as the presence of lymph node metastatic disease.

In the report by Spiller et al., the utility and feasibility of imaging, computer vision and ML to determine patient-derived organoids vital status is reported. By acquiring bright field images at different time points without relying upon vital dyes, authors track the dynamic response of individual organoids to various drugs. In addition, authors report a web-based data visualization tool, called the Organoizer, available for public use.

Abousamra et al. present a Deep Learning workflow that generates Tumor Infiltrating Lymphocytes (TIL) maps to study their abundance and spatial distribution in 23 cancer types. Authors trained three state-of-the-art CNN architectures (namely VGG16, Inception-V4, ResNet-34) with training data from The Cancer Genome Atlas, combining manual annotations from pathologists and computer-generated labels from a first-generation TIL model. It also incorporates automated thresholding to convert model predictions into binary classifications to generate TIL maps.

With the aim to identify putative biomarkers for lung cancer and to elucidate the pathogenesis of this disease, Bahado-Singh et al. combined AI and DNA methylation analysis of circulating cell-free tumor DNA. The study analyzes six AI platforms, including Support Vector ML and Deep Learning, to measure cytosine (CpG) methylation changes across the genome in lung cancer. Training sets and validation sets are generated and 10-fold cross validation performed. To elucidate lung cancer pathogenesis, gene enrichment analysis using g:profiler and GREAT enrichment is done.

Triple-negative breast cancer (TNBC) always requires neoadjuvant chemotherapy (NACT) for a pathological complete response and improved long-term survival. Irajizad et al. previously identified a polyamine biomarker suitable to assess which patient will respond to NACT. In their contribution, Irajizad et al. identified TNBC patients who will be insensitive to NACT, by using ML methods.

Finally, the contribution by Batch et al. aimed to improve the detection of metastatic disease over time from structured radiology reports with the ultimate goal of building and updating a Digital Twin to model long-term prognosis. By exposing prediction models to historical information using Natural Language Processing (NLP), the authors were able to extract and encode relevant features from medical text reports, and use these features to develop, train, and validate models. Over 700 thousand radiology reports were used for model development to predict the presence of metastatic disease. The model uses features from consecutive structured patient text radiology reports. Three models were developed to classify the type of metastatic disease: a simple CNN, a CNN augmented with an attention layer, and Recurrent Neural Network labels. To develop the models, a subset of the reports was curated for ground-truth. Results from the three models were compared (accuracy, precision, recall, and F1-score) to a single-report model previously developed to analyze one report instead of multiple past reports. Results suggest that NLP models can extract cancer progression patterns from multiple consecutive reports and predict the presence of metastatic disease in multiple organs with higher performance when compared with a single-report-based prediction.

In summary, contributions to this special edition highlight how AI will accelerate the advancement of Personalized Medicine and cancer care, by improving patient diagnosis, treatment, and prognosis.

## Author contributions

All authors listed have made a substantial, direct, and intellectual contribution to the work and approved it for publication.
